# PPalign: optimal alignment of Potts models representing proteins with direct coupling information

**DOI:** 10.1186/s12859-021-04222-4

**Published:** 2021-06-10

**Authors:** Hugo Talibart, François Coste

**Affiliations:** Univ Rennes, Inria, CNRS, IRISA, Rennes, France

**Keywords:** Direct coupling analysis, Potts model, Integer linear programming, Proteins, Sequence alignment, Homology, Coevolution

## Abstract

**Background:**

To assign structural and functional annotations to the ever increasing amount of sequenced proteins, the main approach relies on sequence-based homology search methods, e.g. BLAST or the current state-of-the-art methods based on profile Hidden Markov Models, which rely on significant alignments of query sequences to annotated proteins or protein families. While powerful, these approaches do not take coevolution between residues into account. Taking advantage of recent advances in the field of contact prediction, we propose here to represent proteins by Potts models, which model direct couplings between positions in addition to positional composition, and to compare proteins by aligning these models. Due to non-local dependencies, the problem of aligning Potts models is hard and remains the main computational bottleneck for their use.

**Methods:**

We introduce here an Integer Linear Programming formulation of the problem and PPalign, a program based on this formulation, to compute the optimal pairwise alignment of Potts models representing proteins in tractable time. The approach is assessed with respect to a non-redundant set of reference pairwise sequence alignments from SISYPHUS benchmark which have lowest sequence identity (between $3\%$ and $20\%$) and enable to build reliable Potts models for each sequence to be aligned. This experimentation confirms that Potts models can be aligned in reasonable time ($1'37''$ in average on these alignments). The contribution of couplings is evaluated in comparison with HHalign and independent-site PPalign. Although Potts models were not fully optimized for alignment purposes and simple gap scores were used, PPalign yields a better mean $F_1$ score and finds significantly better alignments than HHalign and PPalign without couplings in some cases.

**Conclusions:**

These results show that pairwise couplings from protein Potts models can be used to improve the alignment of remotely related protein sequences in tractable time. Our experimentation suggests yet that new research on the inference of Potts models is now needed to make them more comparable and suitable for homology search. We think that PPalign’s guaranteed optimality will be a powerful asset to perform unbiased investigations in this direction.

## Background

Thanks to sequencing technologies, the number of available protein sequences has considerably increased in the past years, but their functional and structural annotation remains a bottleneck. This task is thus classically performed in silico by scoring the alignment of new sequences to well-annotated homologs. One of the best-known method is BLAST [1], which performs pairwise sequence alignments. The main tools for homology search are now based on profile Hidden Markov Models (pHMMs), which model position-specific composition, insertion and deletion probabilities of each family of homologous proteins. Two well-known software packages using pHMMs are widely used today: HMMER [2] aligns sequences to pHMMs and HH-suite [3] takes it further by aligning pHMMs to pHMMs.

Despite their solid performance, pHMMs are innerly limited by their positional nature. Yet, it is well-known that residues that are distant in the sequence can interact and co-evolve, e.g. due to their spatial proximity, resulting in correlated positions. One can cite for instance experiments of Ranganathan et al. on the WW domain who showed by experimentally testing libraries of artificial sequences of the WW domain that coevolution information is necessary to reproduce the functional properties of native proteins [4].

There have been a few attempts to make use of long-distance sequence information. Menke, Berger and Cowen introduced a Markov Random Field (MRF) approach, SMURF [5], where MRFs generalize pHMMs by allowing dependencies between paired residues in $\beta$-strands to recognize proteins that fold into $\beta$-structural motifs. Their MRFs are trained on multiple structure alignments. A model simplification [6] and heuristics [7] have been proposed to speed up the process. While these methods outperform HMMER [2] in propeller fold prediction, they are limited to sequence-MRF alignment on $\beta$-strand motifs with available structures. Xu et al. [8] proposed a more general method, MRFalign, which performs MRF-MRF alignments using probabilities estimated by neural networks from amino acid frequencies and mutual information. Unlike SMURF, MRFalign handles dependencies between all positions and MRFs are built from multiple sequence alignments.

In addition to these inputs, MRFalign relies on complex scoring functions based on Conditional Neural Fields and Probabilistic Neural Network trained on reference alignments and structural information to optimize the similarity measures of the positional and coupling potentials of the MRF models to be compared. In reported results, PSSM–PSSM and HMM–HMM alignment methods are outperformed by MRFalign in terms of both alignment accuracy and remote homology detection accuracy, notably on mainly beta proteins, showing the potential of using long-distance information in protein sequence alignment.

Meanwhile, a more interpretable type of MRF grounded in the maximum entropy principle led to a breakthrough in the field of contact prediction [9]: the Potts model. This model was brought forward by Direct Coupling Analysis [10], a statistical method to extract direct correlations from multiple sequence alignments. Once inferred on a multiple sequence alignment (MSA), Potts model’s nodes represent positional conservation, and its edges represent direct couplings between positions in the MSA. Unlike mutual information which also captures indirect correlations between positions, Potts models are global models capturing the collective effects of entire networks of correlations through their coupling parameters [11], thus tackling indirect effects and making them a relevant means of predicting interactions between residues. Beyond contact prediction, the positional and the direct coupling information captured by Potts model’s parameters might also be valuable in the context of protein homology search. The idea of using Potts models for this purpose was simultaneously proposed last year at the 2019 *Workshop on Co-evolutionary Methods for the Prediction and Design of Protein Structure and Interactions* by Muntoni and Weigt [12], proposing to align sequences to Potts models, and by us [13], proposing to align Potts models to Potts models in our generic framework for the comparison of protein sequences using direct coupling information named ComPotts.

A method to align a sequence to an hybrid model between Potts model and profile Hidden Markov Model was concurrently proposed by Wilburn and Eddy with Hidden Potts Models [14].

The main computational bottleneck for such approaches is that, due to non-local dependencies, alignment problems involving Potts models are hard. Muntoni and Weigt [15] proposed an approximate message-passing algorithm to align a sequence to a Potts model, while Wilburn and Eddy [14] proposed a method based on importance sampling. We present here PPalign, the alignment method we introduced in ComPotts to optimally align two Potts models representing proteins in tractable time with respect to our Integer Linear Programming (ILP) formulation of the problem. This work builds with an adequate scoring function on the ILP formulation of Wohlers, Andonov, Malod-Dognin and Klau [16, 17] of the distance matrix alignment problem initiated by DALI to perform protein structure alignment [18] and their efficient solver extending itself to real valued pairwise scores their solver for protein structure alignment by Contact Map Overlap (CMO) maximisation [19], a well-studied problem where Linear Programming strategies are known to be efficient [20, 21]. In contrast to these methods using pairwise information from protein structures, in our approach proteins are aligned using pairwise information from protein sequences only. Our method can then be significantly different in not only considering contact or coupling strength information between position pairs but also their coupled amino-acid composition.

This paper fully describe our Potts to Potts model alignment of sequences approach and focus on its performances in terms of alignment quality on remote homologs. In the following sections, we explain our choices for the inference of Potts models and describe the method PPalign for aligning them. To assess the tractability and the quality of alignments by this approach, we extracted 33 non-redundant pairwise reference alignments with a particularly low identity from the manually curated structural alignments database SISYPHUS [22] and randomly split it into a training set of 11 pairs to train our hyperparameters and a test set of 22 pairs on which we compared our results with HHalign’s alignments of pHMMs built on the same input data. On this test set, our method yielded the exact solutions up to a chosen epsilon in tractable time, and outperformed HHalign in terms of alignment quality with an $F_1$ score better on average and significantly better for 5 alignments, suggesting that direct couplings can improve alignment quality of remote homologs.

## Methods

### Inference of Potts models

Potts models are discrete instances of pairwise Markov Random Fields which originate from statistical physics. They generalize Ising models by describing interacting spins on a crystalline lattice with a finite alphabet. In the paper introducing Direct Coupling Analysis, Weigt et al. came up with the idea of applying them to proteins: by building a multiple sequence alignment of a protein sequence and its close homologs and inferring a Potts model on it, one can predict contacts between residues by looking at its parameters [10].

The inference of a Potts model from a set of protein sequences can be formally defined as follows:

Let $S=\{s^n\}_{n=1,\ldots ,N}$ be a set of *N* protein sequences of lengths $l_1,\ldots ,l_N$. A multiple sequence alignment (MSA) of these sequences can be defined as a set of *N* sequences $X=\{x^n\}_{n=1,\ldots ,N}$ on the alphabet of *S* extended with a new gap character ’−’, which all have the same length *L* and such that removing all gaps from a sequence $x^n$ gives $s^n$. By extension, *L* is called the length of the MSA. We denote by *q* the size of the alphabet.

A Potts model with *q* states for MSA *X* can be defined as a statistical model whose probability distribution *P* over all sequences of length *L* maximizes the Shannon entropy ${H(P)=-\sum _{y \in \{1,\ldots ,q\}^L} P(y) \log P(y)}$ and generates the empirical single and double frequencies of the MSA as marginals:

1 $$\begin{aligned} \forall i& = {} 1,\ldots ,L, \forall a=1,\ldots ,q, \sum _{\begin{array}{c} y \in \{1,\ldots ,q\}^L \\ y_i=a \end{array}} P(y) = f_i(a) = \frac{1}{N} \sum _{n=1}^N \delta \left( x_i^n,a\right) \end{aligned}$$

2 $$\begin{aligned} \forall i,j& = {} 1,\ldots ,L, \forall a, b=1,\ldots ,q, \sum _{\begin{array}{c} y \in \{1,\ldots ,q\}^L \\ y_i=a, y_j=b \end{array}} P(y) = f_{ij}(a,b) & = {} \frac{1}{N} \sum _{n=1}^N \delta (x_i^n,a) \delta \left( x_j^n,b\right) \end{aligned}$$

This probability distribution has the following form:

3 $$\begin{aligned} P(X=x|{\boldsymbol{v}}, {\boldsymbol{w}}) = \frac{1}{Z} \exp \left( - {\mathcal {H}}(x|{\boldsymbol{v}}, {\boldsymbol{w}}) \right) \end{aligned}$$

where *Z* is a normalization constant : $Z=\sum _{y \in \{1, \ldots , q\}^L} \exp \left( - {\mathcal {H}}(y|{\boldsymbol{v}}, {\boldsymbol{w}}) \right)$ and ${\mathcal {H}}$ is an energy function defined as

4 $$\begin{aligned} {\mathcal {H}}(x|{\boldsymbol{v}}, {\boldsymbol{w}}) = -\left( \sum _{i=1}^L v_i(x_i) + \sum _{i=1}^{L-1} \sum _{j=i+1}^L w_{ij}(x_i,x_j) \right) \end{aligned}$$

where the parameters $({\boldsymbol{v}},{\boldsymbol{w}})$ that define a Potts model are the ones that maximize the likelihood of the sequences in the MSA *X*:

5 $$\begin{aligned} {\boldsymbol{v}}, {\boldsymbol{w}}& = {} {\mathop {{{\,\mathrm{argmax}\,}}}\limits _{v,w}} \prod _{n=1}^N P \left( X=x^n|v,w\right) \\ &= {\mathop {{{\,\mathrm{argmax}\,}}}\limits _{{v,w}}} \prod _{n=1}^N \frac{1}{Z} \exp \left( \sum _{i=1}^L v_i(x^n_i)  + \sum _{i=1}^{L-1} \sum _{j=i+1}^L w_{ij}\left( x^n_i,x^n_j\right) \right) \end{aligned}$$

These parameters can be assigned a practical interpretation:

- ${\boldsymbol{v}} = \{v_i\}_{i=1,\ldots ,L}$ are positional parameters termed “fields”. Each $v_i$ is a real vector of length *q* where $v_i(a)$ is related to the propensity of letter *a* to be found at position *i*.
- ${\boldsymbol{w}} = \{w_{ij}\}_{i,j=1,\ldots ,L}$ are pairwise coupling parameters. Each $w_{ij}$ is a $q \times q$ real matrix where $w_{ij}(a,b)$ quantifies how compatible letters *a* and *b* are when found at positions *i* and *j*.

**Fig. 1:**
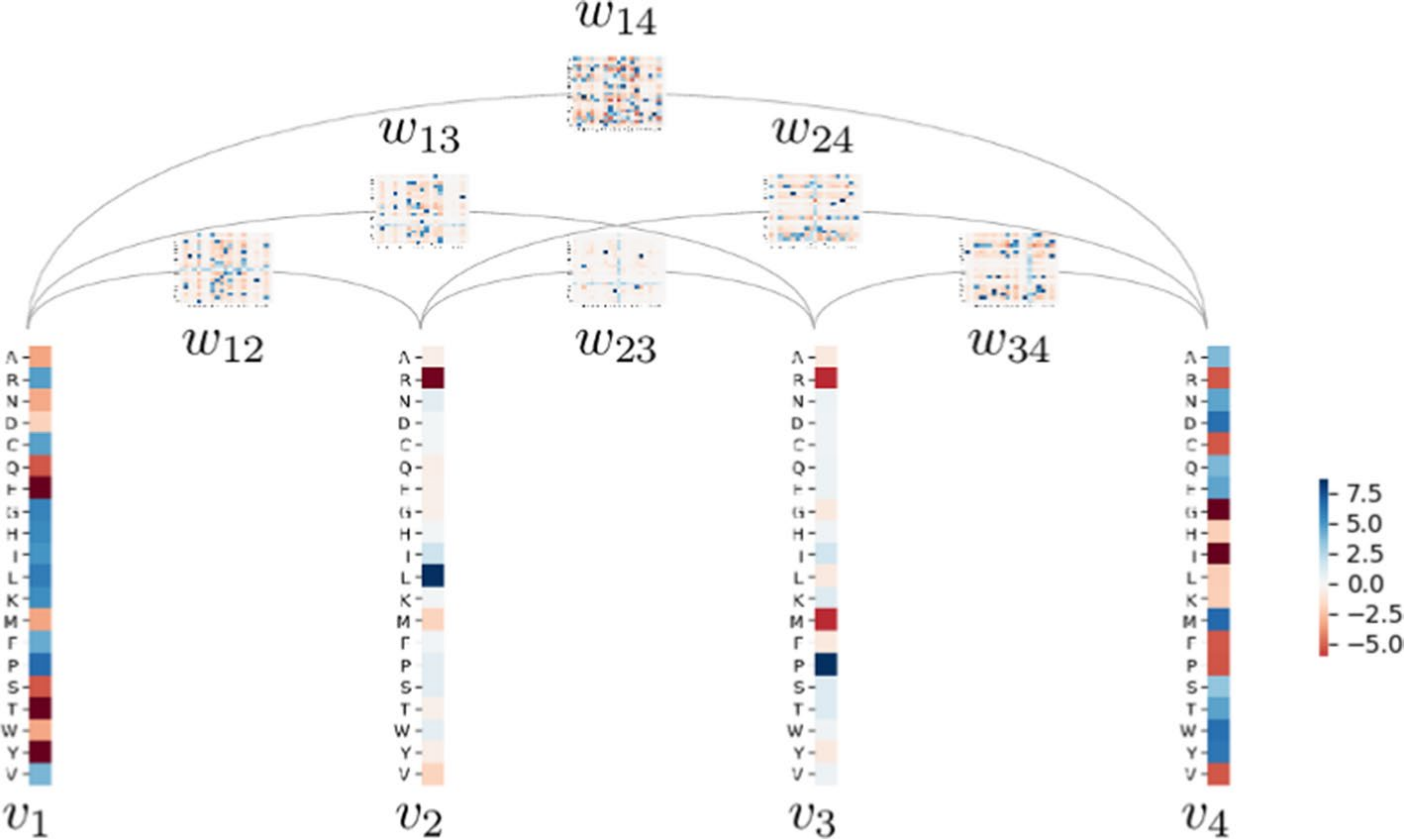
Example of Potts model representing a MSA of length 4. Each column in the MSA is associated with a field vector $v_i$ of length $q=20$ where each $v_i(a)$ is a real value weighting positively or negatively the occurrence of letter *a* at position *i*. Each pair of positions (*i*, *j*) is associated with a $q \times q$ coupling matrix $w_{ij}$ where $w_{ij}(a,b)$ are real values weighting positively or negatively the co-occurrence of letters *a* and *b* respectively at position *i* and *j*

An illustration of Potts model is given Fig. 1.

These parameters are unique up to a *gauge invariance*: Eqs. (1) and (2) are not independent, implying that the probability distribution remains unchanged under the following transformation:

6 $$\begin{aligned} {\left\{ \begin{array}{ll} w_{ij}(a,b) \leftarrow w_{ij}(a,b) + K_{ij}(a) + K_{ji}(b)\\ v_i(a) \leftarrow v_i(a) + C_i - \sum _{j \ne i} (K_{ij}(a)+K_{ji}(a)) \end{array}\right. } \end{aligned}$$

where $K_{ij}$ and $C_i$ are arbitrary values. In our case, this indeterminacy is fixed with the widely-used zero-sum gauge:

7 $$\begin{aligned} \forall i, j, b, \sum _a w_{ij}(a,b) = \sum _a v_i(a) = 0 \end{aligned}$$

In practice, maximizing the likelihood would require the computation of the normalization constant *Z* at each step, which is computationally intractable. Among the several approximate inference methods that have been proposed [11, 23–26], we opted here for pseudo-likelihood maximization since it was proven to be a consistent estimator in the limit of infinite data [27, 28] within reasonable time. Furthermore, since our goal is to align Potts models, we need the inferrence to be geared towards similar models for similar MSAs, which is not what inference methods were initially designed for. In an effort towards inferring canonical Potts models, we have chosen here to use CCMpredPy [29], a recent Python-based version of CCMpred [30] which, instead of using the standard $L_2$ regularization prior ${R(v,w) = \lambda _v \left\Vert v\right\Vert _2^2 + \lambda _w \left\Vert w\right\Vert _2^2}$, allows us to use a smarter prior on *v*:

8 $$\begin{aligned} R(v,w) = \lambda _v \left\Vert v-v^*\right\Vert _2^2 + \lambda _w \left\Vert w\right\Vert _2^2 \end{aligned}$$

where $$v^*$$ obeys

9 $$\begin{aligned} \frac{\exp \left( v^*_i(a)\right) }{\sum _{b=1}^q \exp \left( v^*_i(b)\right) } = f_i(a) \end{aligned}$$

which yields the correct probability model if no columns are coupled, i.e. $P(x|v,w) = \prod _{i=1}^L P(x_i)$. Our intuition is that positional parameters should explain the MSA as much as possible and only necessary couplings should be added.

### From a protein sequence to a Potts model

Unlike homology detection methods based on simple pairwise sequence alignment such as BLAST, as HHalign our method explicitly considers sequence conservation and variability around sequences to be compared by modeling each sequence and its retrieved close homologs, with the addition of coupling information in our case. This implies that the quality of the alignment will be dependent on the quality of the MSAs of close homologs built for each sequence. In this paper, based on CCMpred’s recommendations [31], for each sequence we run HHblits [3] v3.03 with the following parameters:

${\texttt {-maxfilt~100000}}~{\texttt {-realign}}\_{\texttt {max}}~{\texttt {100000 -all -B 100000 -Z 100000 -n 3 -e 0.001}}$ on Uniclust30 [32] (08/2018 release), and then process the output by:

- filtering at $80\%$ identity using HHfilter
- taking the first 1000 sequences
- removing all columns with $>50\%$ gaps using trimal [33]

The resulting MSA is inputted to CCMpredy [29] using default parameters to infer a Potts model, and trimmed positions *i* (with $>50\%$ gaps in the input MSA) are re-inserted in the model with positional parameters at position *i* set to background fields defined using frequencies $f_0$ given by [34]

10 $$\begin{aligned} v_0(a) = \log f_0(a) - \frac{1}{q} \sum _{b=1}^q \log f_0(b) \end{aligned}$$

and pairwise coupling parameters with position *i* set to:

11 $$\begin{aligned} \forall j, a, b, w_{ij}(a,b) = 0 \end{aligned}$$

### Parameter rescaling strategy

Since existing Potts model inference methods were specifically designed for the prediction of co-evolving position pairs, inferred parameters might not be ideally suited for Potts model comparison. This section describes two strategies implemented to compensate for these shortcomings.

#### Lessening the effect of small sample variations on the positional parameters

Since field parameters *v* are linked to single frequencies through a logarithmic relation [see Eq. (9)], any noise in the presence of small probabilities can have a great impact on the model parameters. This has a dramatic effect on the scoring function we use for pairwise Potts model alignment since the sign of each parameter directly determines the sign of their similarity score (see next section). To lessen the effects of sampling variations, we apply additive smoothing to the softmax probability distribution $p_i$ associated with each $v_i$.

More formally, a standard softmax probability distribution $p_i$ is extracted for each positional parameter $v_i$:

12 $$\begin{aligned} \forall a \, p_i(a) = \frac{\exp (v_i(a))}{\sum _{b=1}^{q} \exp (v_i(b))} \end{aligned}$$

It is then smoothed towards a uniform distribution so that very low probabilities are more homogenized:

13 $$\begin{aligned} \tilde{p_i}(a) = (1-\tau _v) p_i(a) + \frac{\tau _v}{q} \end{aligned}$$

where $\tau _v$ is a parameter controlling the amount of additive smoothing used. Final smoothed parameters $\tilde{v_i}(a)$ are retrieved by inverting the softmax function using the fact that $\sum _{a=1}^{q} v_i(a) = 0$ according to CCMpredPy’s gauge choice:

14 $$\begin{aligned} \tilde{v_i}(a) = \log \tilde{p_i}(a) - \frac{1}{q} \sum _{b=1}^{q} \log \tilde{p_i}(b) \end{aligned}$$

Summing up in one formula, each parameter $v_i(a)$ of the inferred Potts model is smoothed using the following function:

15 $$\begin{aligned} \tilde{v_i}(a) &= \log \left( (1-\tau _v) \frac{\exp (v_i(a))}{\sum _{b=1}^{q} \exp (v_i(b))} + \frac{\tau _v}{q} \right) \\ &- \frac{1}{q} \sum _{c=1}^{q} \log \left( (1-\tau _v) \frac{\exp (v_i(c))}{\sum _{b=1}^{q} \exp (v_i(b))} + \frac{\tau _v}{q} \right) \end{aligned}$$

#### Diminishing contributions of anti-correlations

In theory, coupling values inside a $w_{ij}$ matrix are supposed to deviate positively or negatively from 0 to reflect a (direct) correlation or anti-correlation. In practice however, while input data can be sufficient to assert that two letters *a* and *b* are likely to be found together at positions *i* and *j*, deducing that they should not be found together at positions *i* and *j* requires more examples to have sufficient countings on all pairs of *a* and *b*. Considering that our data set is limited, a large number of spurious anti-correlations can arise from a mere lack of data.

Since positive correlations are more likely to be supported by available training sample than negative ones, our approach here is to skew the coupling value distribution inside each $w_{ij}$ matrix to favor higher, positive values.

To do this, we extract each coupling matrix probability distribution as for the fields, only with a different softmax base $\beta _w$, chosen so that the extracted distribution is skewed towards higher probabilities and, as for the fields, smooth it towards a uniform distribution to lessen noise, which gives:

16 $$\begin{aligned} \tilde{w_{ij}}(a,b) = \frac{1}{\beta _w} \left( \log \left( (1-\tau _w) \frac{\exp (\beta _w w_{ij}(a,b))}{\sum _{c=1}^{q} \sum _{d=1}^{q} \exp (\beta _w w_{ij}(c,d))} + \frac{\tau _w}{q^2} \right) \right.   \\ \left. - \frac{1}{q^2} \sum _{e=1}^{q} \sum _{f=1}^{q} \log \left( (1-\tau _w) \frac{\exp (\beta _w w_{ij}(e,f))}{\sum _{c=1}^{q} \sum _{d=1}^{q} \exp (\beta _w w_{ij}(c,d))} + \frac{\tau _w}{q^2} \right) \right) \end{aligned}$$

Using this smoothing scheme on each input Potts model make them more comparable since the most significant information stands out while sampling variations are tuned down.

This strategy was implemented to compensate for the impossibility to add pseudo-counts when inferring models with methods based on pseudo-likelihood, such as CCMpredPy, which we selected for its smart prior on the field parameters. For future experiments, we are hoping to find an inference method with the same prior and allowing us to add pseudo-counts on the single and the double frequencies. This smoothing strategy will then probably no longer be needed.

### Alignment of Potts models

This section introduces our method for aligning two Potts models. The function we designed to score a given alignment is described and constraints ensuring that the alignment is proper are added as in Wohlers et al. [17], resulting in an Integer Linear Programming formulation that can be optimized using their efficient solver.

#### Scoring function

Basically, the best alignment between two Potts models $A=({\boldsymbol{v}}^A,{\boldsymbol{w}}^A)$ and $B=({\boldsymbol{v}}^B,{\boldsymbol{w}}^B)$ of lengths $L_A$ and $L_B$ is defined as the alignment which maximizes the similarity between aligned fields and aligned couplings. Formally, this means finding the values of the binary variables $x_{ik}$ where $x_{ik}=1$ iff position *i* in Potts model *A* is aligned with position *k* in Potts model *B* so as to maximize:

17 $$\begin{aligned} s(A,B) = \sum _{i=1}^{L_A} \sum _{k=1}^{L_B}s_v\left( v_i^A, v_k^B\right) x_{ik} &+ \alpha _w \sum _{i=1}^{L_A-1} \sum _{j=i+1}^{L_A} \sum _{k=1}^{L_B-1} \sum _{l=k+1}^{L_B} s_w\left( w^A_{ij}, w^B_{kl}\right) y_{ikjl} \end{aligned}$$

where $y_{ikjl}=x_{ik}x_{jl}$, $s_v(v_i^A,v_k^B)$ and $s_w(w_{ij}^A,w_{kl}^B)$ are similarity scores, respectively between positional parameters $v_i^A$ and $v_k^B$ and coupling parameters $w_{ij}^A$ and $w_{kl}^B$, and $\alpha _w$ is a coefficient ensuring proper balance between positional and coupling score.

To measure the similarity between vectors, the scalar product is a natural candidate. We propose thus to measure the similarity $s_v(v_i^A, v_k^B)$ between field parameters using:

18 $$\begin{aligned} \langle v_i^A, v_k^B \rangle = \sum _{a=1}^q v_i^A(a) v_k^B(a) \end{aligned}$$

and to measure the similarity $s_w(w^A_{ij}, w^B_{kl})$ between coupling parameters by the extension of the scalar product to matrices, the Frobenius inner product:

19 $$\begin{aligned} \langle w_{ij}^A, w_{kl}^B \rangle = \sum _{a=1}^q \sum _{b=1}^q w_{ij}^A(a,b) w_{kl}^B(a,b) \end{aligned}$$

Note that this scoring function for two Potts models naturally generalizes the score of a sequence *x* for a given Potts model since its energy can be computed as:

20 $$\begin{aligned} {\mathcal {H}}(x|{\boldsymbol{v}},{\boldsymbol{w}}) = - \left( \sum _{i=1}^L v_i(x_i) + \sum _{i=1}^{L-1} \sum _{j=i+1}^L w_{ij}(x_i,x_j) \right) &= - \left( \sum _{i=1}^L \langle v_i, e_{x_i} \rangle + \sum _{i=1}^{L-1} \sum _{j=i+1}^L \langle w_{ij}, e_{x_i x_j} \rangle \right) \end{aligned}$$

where

- $e_{x_i}$ is the vector defined by $\forall a \in [1..q], e_{x_i}(a) = \delta (a, x_i)$
- $e_{x_ix_j}$ is the matrix defined by $\forall (a,b) \in [1..q]^2, e_{x_ix_j}(a,b) = \delta (a,x_i)\delta (b,x_j)$

Inspired by sequence alignment methods which use log-odds ratios to compute their scores with respect to a background model, we remove the background field $v_0$ defined in Eq. (10) to each field vector before computing the scalar product. The actual similarity score between two positional parameters $v_i^A$ and $v_k^B$ used in this paper is thus:

21 $$\begin{aligned} s_v\left( v_i^A,v_k^B\right) = \langle v_i^A - v_0, v_k^B - v_0 \rangle \end{aligned}$$

while the similarity score between two coupling parameters $w_{ij}^A$ and $w_{kl}^B$ remains:

22 $$\begin{aligned} s_w(w_{ij}^A, w_{kl}) = \langle w_{ij}^A, w_{kl}^B \rangle \end{aligned}$$

#### Optimizing score with respect to constraints

Naturally, the scoring function should be maximized with respect to constraints ensuring that the alignment is proper. In that perspective, we build on the work of Wohlers et al. [17], initially dedicated to protein structure alignment, to propose an Integer Linear Programming formulation for the Potts model alignment problem.

Let us first introduce necessary definitions and notations following [17] to define a proper alignment.

The *alignment graph* of two Potts models *A* and *B* of lengths $L_A$ and $L_B$ is a $L_A \times L_B$ grid graph where rows (from bottom to top) represent positions in *A* and columns (from left to right) represent positions in *B*. A node *i*.*k* in the alignment graph represents the alignment of node *i* from Potts model *A* and node *k* from Potts model *B*. Directed edges (*i*.*k*, *j*.*l*) are drawn for $i<j$ and $k<l$. In this framework, an alignment of *n* positions in the two Potts models is represented by a set of nodes $\{i_1.k_1, \ldots , i_n.k_n\}$ where $i_1< \cdots < i_n$ and $k_1< \cdots < k_n$, termed *increasing path*.

In order to properly set constraints on the alignment, two additional node sets are defined: $\text {row}_{ik}(j)$ (resp. $\text {col}_{ik}(l)$) is the maximal set of nodes in the alignment graph that are tails of edges with head at *i*.*k* or heads of edges with tail at *i*.*k*, that contain at least one node at row *j* (resp. column *l*), and that *mutually contradict*, i.e. no two of them lie on an increasing path.

To cast the alignment problem into an ILP, binary variables $x_{ik}$ are assigned to each node *i*.*k* in the alignment graph, with $x_{ik}=1$ if position *i* in Potts model *A* and position *k* in Potts model *B* are aligned, and similarly a binary variable $y_{ikjl}$ is assigned to each edge in the alignment graph where $y_{ikjl}=1$ if edge (*i*.*k*, *j*.*l*) is activated.

Given notations above, the alignment of two Potts models *A* and *B* of lengths $L_A$ and $L_B$ and parameters $(v^A, w^A)$, $(v^B,w^B)$ can be formulated as the following Integer Linear Programming problem:

23 $$\begin{aligned}&\text {max} \quad \sum _{i=1}^{L_A} \sum _{k=1}^{L_B} s_v\left( v_i^A, v_k^B \right) x_{ik} + \alpha _w \sum _{i=1}^{L_A-1} \sum _{j=i+1}^{L_A} \sum _{k=1}^{L_B-1} \sum _{l=k+1}^{L_B} s_w\left( w_{ij}^A, w_{kl}^B\right) y_{ikjl} \end{aligned}$$

24 $$\begin{aligned}&\text {s.t.} \quad x_{ik} \ge \sum _{r.s \in \textit{row}_{ik}(j)} y_{ikrs} \quad j \in [i+1,L_A], i \in [1,L_A-1], k \in [1, L_B-1] \end{aligned}$$

25 $$\begin{aligned}&x_{ik} \ge \sum _{r.s \in \textit{col}_{ik}(l)} y_{ikrs} \quad l \in [k+1,L_B], i \in [1, L_A-1], k \in [1,L_B-1] \end{aligned}$$

26 $$\begin{aligned}&x_{ik} \ge \sum _{r.s \in \textit{row}_{ik}(j)} y_{rsik} \quad j \in [1,i-1], i \in [2,L_A], k \in [2,L_B] \end{aligned}$$

27 $$\begin{aligned}&x_{ik} \ge \sum _{r.s \in \textit{col}_{ik}(l)} y_{rsik} \quad l \in [1,k-1], i \in [2,L_A], k \in [2,L_B] \end{aligned}$$

28 $$\begin{aligned}&x_{ik} \le \sum _{\begin{array}{c} r.s \in \textit{row}_{ik}(j) \\ s(A_{ri},B_{sk}) \le 0 \end{array}} (y_{rsik}-x_{rs}) +1 \quad j \in [1,i-1], i \in [2,L_A], k \in [2,L_B] \end{aligned}$$

29 $$\begin{aligned}&\sum _{l=1}^k x_{il} + \sum _{j=1}^{i-1} x_{jk} \le 1 \quad i \in [1,L_A], k \in [1,L_B] \end{aligned}$$

30 $$\begin{aligned}&x, y \textit{ binary} \end{aligned}$$

Constraints (24) and (25) prevent edges from activating if their tails are not activated and ensure that heads of edges with a common tail do not contradict, and constraints (26) and (27) denote the reverse situation. Constraint (28) ensures that edges are activated if their heads and tails are activated (this constraint is necessary since similarity scores can be negative). Finally, constraint (29) ensures that the nodes lie on an increasing path.

A major asset of the solver is that it can yield the exact solution of this ILP, or a solution within a chosen epsilon range of the exact one, in tractable time. Desired precision of the optimization can be set by the parameter $\epsilon$, ensuring that ${\frac{2(\text {UB}-\text {LB})}{s(A,A)+s(B,B)} \le \epsilon }$ where $\text {UB}$ and $\text {LB}$ are the upper and lower bounds guaranteed by the solver for the solution, to avoid unnecessary optimization steps (the precision can be sufficient for the task) and speed up the search (often the last optimization steps only contribute to tighten the bounds while the optimal solution is already found).

#### Gap cost and offset

As in [17], an affine gap cost function can be added to the score function to account for insertions and deletions in the sequences, with the appropriate choice of a gap open and a gap extend penalties.

Furthermore, as in most profile-profile methods [35], in order to prevent our method from greedily aligning every position, we penalize each aligned pair with a fixed negative offset hyperparameter.

### Data

To evaluate PPalign and the contribution of distant dependencies, we focused on reference alignments based on structures with low sequence identity. We opted for SISYPHUS database [22] since it provides manually curated structural alignments for proteins with non-trivial relationships. Our data set was built as follows:

- From each multiple sequence alignment in SISYPHUS, every possible pairwise sequence alignment with a sequence identity lower than $20\%$ was extracted (we set a low sequence identity threshold to focus on harder targets)
- For each sequence in each of these extracted pairwise reference alignments, we attempted to build a Potts model with the workflow previously described. Sequences that had less than 1000 $80\%$ non-redundant homologs were discarded to focus on sequences with sufficient co-evolution signal. Due to CCMpredPy memory consumption, trimmed MSAs whose length was longer than 200 also had to be discarded.
- Finally, for each reference multiple sequence alignment in SISYPHUS with more than two of such eligible sequences, a reference sequence pair was randomly selected. This last steps discards many alignment pairs but ensures that no multiple sequence alignment biases the results.

This resulted in a set of 33 non-redundant reference pairwise alignments which was randomly split into a train set of 11 alignments on which our hyperparameters were trained (see Table 1) and a test set of 22 target alignments (see Table 2).

**Fig. 2:**
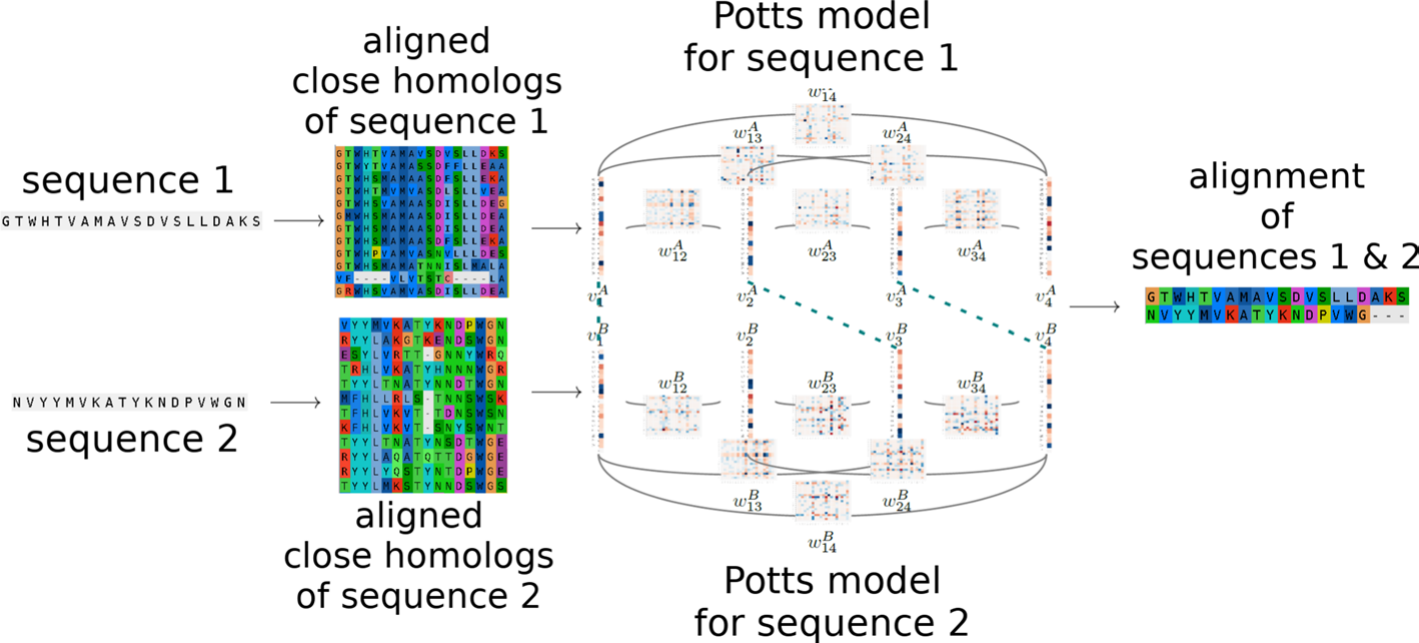
Potts to Potts alignment of two sequences workflow. Coevolution information is obtained for each sequence by retrieving close homologs, and Potts models are inferred on the corresponding multiple sequence alignments. PPalign computes the optimal alignment of the two Potts models, thereby providing an alignment of the two initial sequences

**Fig. 3:**
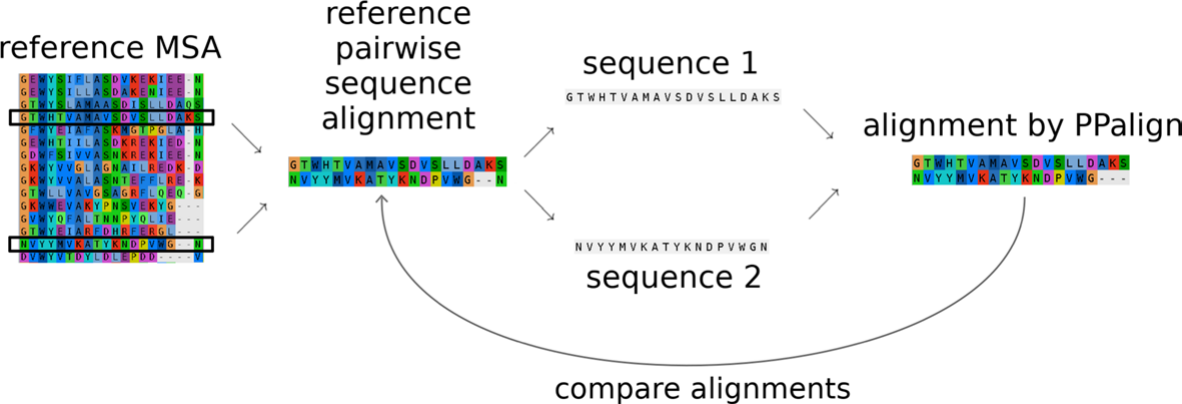
Overview of evaluation procedure for a reference MSA. A reference pairwise sequence alignment is (randomly) extracted from a reference MSA in SISYPHUS. The two sequences are then aligned by PPalign with the workflow previously introduced, and the alignment is compared with the reference alignment in terms of precision, recall and $F_1$ score

The overall workflow to align two protein sequences with our method and the evaluation procedure for each reference MSA are respectively summarized in Figs. 2 and 3.

**Table 1:** Training set

MSA	Sequences	Sequence identity (%)
AL10050464	1r5bA_559_659, 1r5bA_470_549	3.85
AL00053697	1vimA_36_164, 1iatA_334_500	4.04
AL00063412	1bccA_34_201, 1ezvB_236_357	5.59
AL00051306	1ay9A_51_137, 1b12A_81_302	6.28
AL00052113	1kzyC_1731_1838, 1in1A_853_916	8.60
AL10069117	1kncA_13_172, 2gmyA_14_141	9.09
AL00050815	1i4uA_33_167, 1np1A_21_166	10.00
AL00054790	1vig_10_72, 1k1gA_136_223	11.36
AL00054403	4monA_6_47, 1roaA_23_119	13.33
AL00048098	1cmzA_90_199, 1omwA_54_168	13.91
AL00089800	1p6oA_10_147, 1wkqA_2_150	17.88

**Table 2:** Test set

MSA	Sequences	Sequence identity (%)
AL00050475	1ci0A_43_200, 1uscA_12_145	3.61
AL00050692	1uheA_11_87, 1q16A_1084_1225	4.14
AL10050815	1exsA_17_124, 1qftA_27_139	5.04
AL10050875	1rbp_19_140, 1hms_3_131	5.19
AL00050715	1dfuP_2_94, 1qtqA_340_541	5.22
AL00055723	1tu1A_1_140, 1v2bB_18_186	5.81
AL00050799	1pklA_88_180, 1o65A_12_173	6.02
AL00074653	1tolA_151_213, 1ihrA_172_230	6.15
AL10063410	1qf6A_68_223, 1hr6B_48_215	6.29
AL00053335	1ri5A_51_291, 1nv8A_106_279	7.43
AL10050155	1k32A_764_851, 1lcyA_228_321	9.62
AL10050335	1h9mA_5_141, 1v43A_247_366	10.22
AL10074933	1k32A_763_852, 1te0A_257_349	10.68
AL00052141	1mwiA_9_163, 1oe4A_87_277	11.48
AL20089447	1z0rA_8_48, 1n0gA_33_142	12.93
AL00047241	1tjoA_29_171, 1lb3A_15_153	13.01
AL00054814	1egaB_197_282, 1hh2P_199_275	13.40
AL00050021	1jm1A_57_211, 1nykA_54_191	14.61
AL00047861	1m12A_3_74, 1n69B_2_73	15.38
AL00052441	1c30A_7_127, 1w93A_59_184	15.38
AL00054407	1eqkA_11_95, 2ch9A_38_144	15.74
AL00052787	5pnt_5_155, 1jl3A_3_137	17.72

### Alignment evaluation metrics

Alignment quality with respect to SISYPHUS’ reference alignments is assessed by computing alignment precision:

31 $$\begin{aligned} P=\frac{{\# \,correctly\,aligned\,pairs}}{{\# \,aligned\, pairs \,in \,computed \,alignment}} \end{aligned}$$

and recall:

32 $$\begin{aligned} R=\frac{{\# \,correctly \,aligned \,pairs}}{{\# \,aligned\, pairs \,in \,reference \,alignment}} \end{aligned}$$

using Edgar’s qscore program [36] v2.1, and $F_1$ score:

33 $$\begin{aligned} F_1=\frac{2PR}{P+R} \end{aligned}$$

### PPalign’s hyperparameters

PPalign’s hyperparameters were optimized in a supervised fashion on the 11 alignments from the training set using Hyperopt library [37] to maximize the $F_1$ score. This process showed to be excessively time-consuming, Hyperopt being unable to show a convergence on the choice of the parameters after one month. In order to reduce the hyperparameter search space and speed up the convergence of this process, we had to arbitrarily set some parameters after some trials on the training set: precision $\epsilon$ was set to 0.02, $\tau _v$ and $\tau _w$ from Eqs. (15) and (16) were both set to 0.4 and the gap extend penalty was set to 0. In accordance with the expected NP-hardness of the pairwise Potts model alignment problem, time needed to find optimal alignment could be very long for some sets of parameters and even exceed the 6 hours time-out we set. We observed yet that good alignments were usually already found in less than 1 minute and decided to set the time-out by alignment to this value to speed-up more the optimisation of the remaining parameters by Hyperopt, which yielded the following values:

- Gap open penalty: 13
- Coupling contribution coefficient $\alpha _w$: 6
- Softmax base $$\beta _w$$: 8.0
- Offset $$\gamma$$: 1.0

### Other methods to be compared

In this experiment, we compared the results of PPalign with HHalign, the core alignment method of the state-of-the-art remote homology detection method HHsearch. We ran HHalign v3.0.3 with default options to align pHMMs built with HHmake with default options from the MSAs used to infer Potts models (except for the trimming of the positions with $>50\%$ gaps since pHMMs handle well insertions and deletions).

To assess the contribution of direct couplings in sequence alignment, we also used PPalign to compute alignments of independent-site Potts models (i.e. Potts models where positions are assumed to be independent, thus without coupling parameters) with the same hyperparameters (termed “*independent-site PPalign*”).

We also ran BLASTp v2.9.0+ without E-value cutoff on the sequences truncated as in our training MSAs to provide an indication on the sequences’ similarity.

## Results

### Tractable computation time

We examined the computation times of PPalign, independent-site PPalign and HHalign, considering the time they took to align the models (and not the steps to build them, that can be done offline) of the sequence pairs from the test set. Experiments were run on a Debian9 virtual machine with 4 VCPUs (2.3 GHz) and 8 GB RAM. The timeout for each alignment was set to 6 hours.

**Fig. 4:**
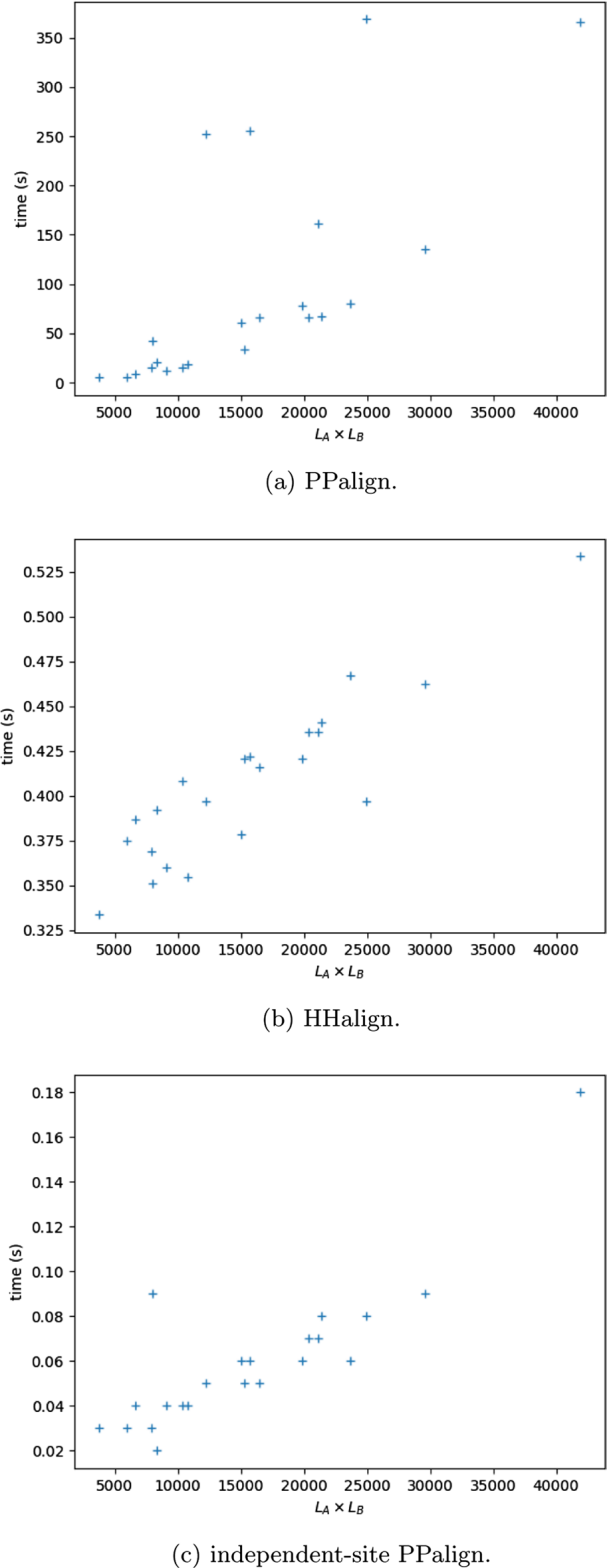
Time for aligning models of lengths $L_A$ and $L_B$ for sequence pairs from test set

The first result is that all the alignments could be computed by PPalign in running times ranging from 5 seconds to 6 minutes, with an average of 1 min 36. Figure 4a plots the running times with respect to the lengths of the models to align. It shows that most problems (17/22) are easily solved and that running time for these problems increases gently with the lengths of the models, while a few (5/22) other problems stand out from this majority trend but are still solved in a few minutes.

When couplings are not considered, the problem is fundamentally easier and running times of HHalign and independent-site PPalign are significantly faster than PPalign: both programs were able to compute each optimal positional alignment in less than 1 second. The running times of HHalign and independent-site PPalign are plotted in Fig. 4b, c . The two plots are not completely comparable since time needed to load the models is here included for HHalign and not for independent-site PPalign, but they illustrate the difference between the dynamic programming approach of HHalign, with a steady running time increment with the length of the models, and the Integer Linear Programming optimization approach of independent-site PPalign, showing here 2 outliers with respect to the general tendency.

### Alignment quality

Alignment quality was assessed by comparing the alignment obtained by the different methods for the 22 sequences pairs in the test set to their reference alignment.

Overall, PPalign achieves a better $F_1$ score than HHalign (0.600 versus 0.578) with a better recall (0.613 vs 0.533) but a lower precision (0.587 vs 0.661), outperforming it in 12 out of the 22 alignments. BLAST only aligned 4 out of the 22 pairs, yielding an average $F_1$ score of 0.113.

**Fig. 5:**
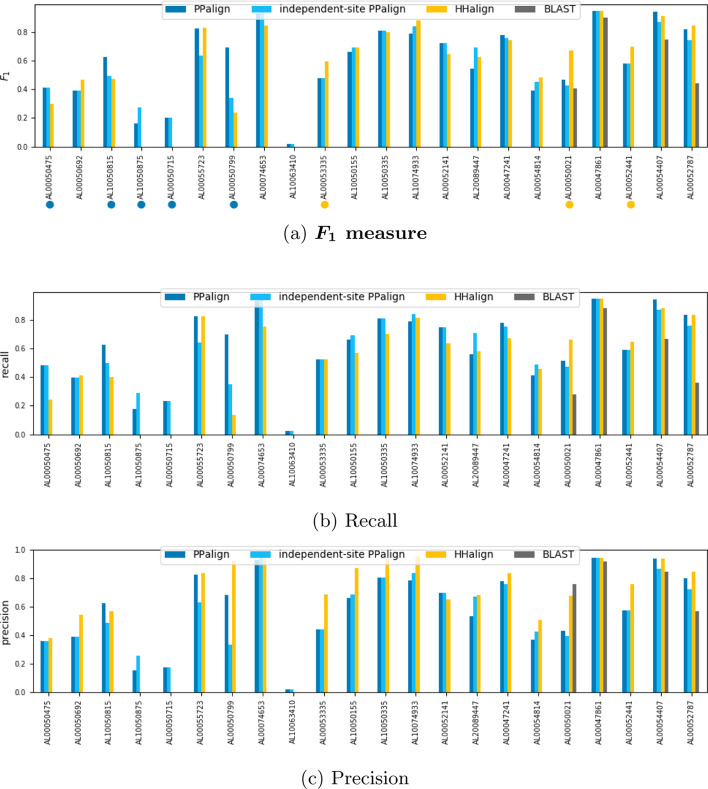
Quality of the alignments computed by PPalign, independent-site PPalign, HHalign and BLAST with respect to target reference alignments in test set (ordered by increasing percentage of sequence identity)

**Fig. 6:**
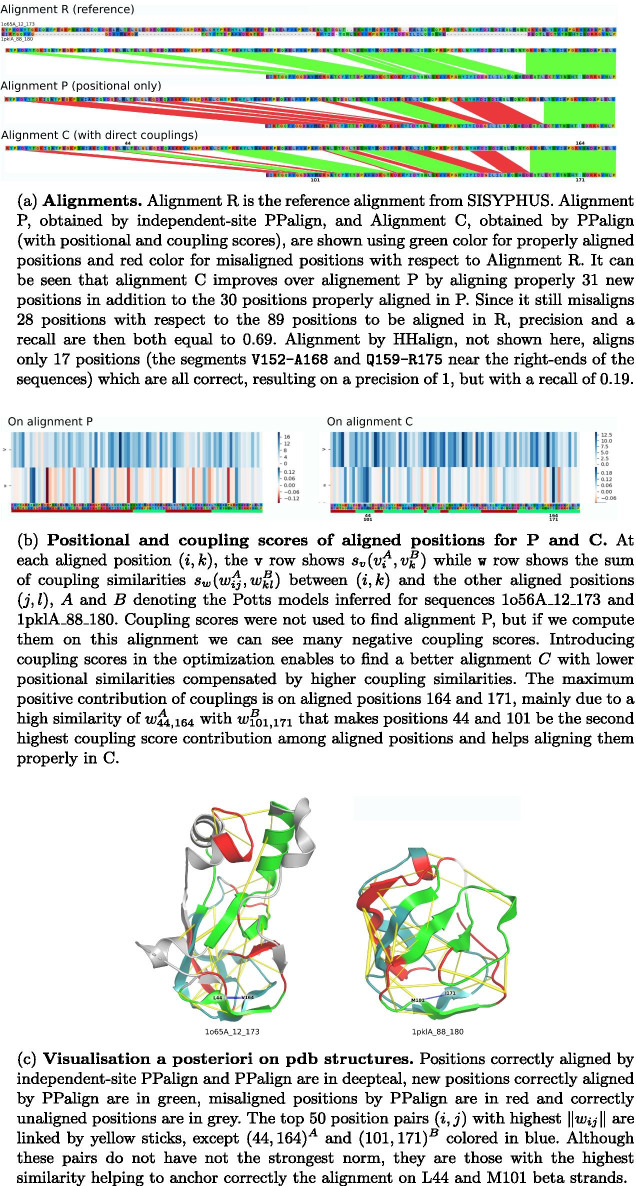
Illustration of the contribution of couplings for the alignment of 1o65A_12_173 and 1pklA_88_180 sequences

Results for each sequence pair of the test set are displayed in Fig. 5 and one example where couplings were particularly helpful in the alignment is discussed Fig. 6.

In most cases, PPalign and HHalign yield similar $F_1$ scores (with less than 0.1 difference), except for 8 sequence pairs. 5 of them, marked by blue dots in the Fig. 5a, are significantly better aligned by PPalign: AL00050475, AL00050692, AL10050875, AL00050715 and AL00050799 which are among the 7 alignments with the smallest percentage of sequence identity with respectively $3.61\%$, $5.04\%$, $5.19\%$, $5.22\%$ and $6.02\%$. AL10050875 and AL00050715 are part with AL10063410 of the three sequence pairs that HHalign fails completely to align, yielding small and incorrect alignments with an $F_1$ score of 0. On AL10063410, PPalign also failed, but on AL10050875 and AL00050715 it was able to do a bit better than HHalign by correctly aligning in each case roughly a fifth of the target alignment while still being wrong on the four other fifths. On AL00050475 and AL00050692, PPalign successfully retrieves about half of the target alignments when HHalign was retrieving only respectively a fifth and a third of it. The contribution of the coupling parameters is particularly noticeable for AL00050799, PPalign correctly retrieving almost $70\%$ of the alignment while HHalign retrieves only $20\%$ of it (see detailed analysis in Fig. 6).

PPalign is significantly outperformed by HHalign on 3 pairs, marked by yellow dots in Fig. 5b. On AL00053335 ($7.43\%$ sequence identity), PPalign suffers from its tendency to align too many positions: like HHalign it correctly aligns half of the target alignment, but it proposes a longer alignment than HHalign, making its precision drop to around $40\%$ when HHalign stays around $60\%$. The two other pairs are AL00050021 and AL00052441 with respectively $14.61\%$ and $15.38\%$ sequence identity allowing HHalign to correctly align $60\%$ of the target alignment. On AL00052441, PPalign correctly aligns more than $50\%$ of the target alignment but the main difference comes here again from the precision (0.58 vs 0.81). Results on AL00050021 are clearly in favour of HHalign with an $F_1$ score of 0.6 compared to 0.4 for PPalign and can be explained by the extremely gappy MSAs used to build the models (more than $\frac{1}{3}$ positions in the reference alignment were trimmed).

Interestingly, PPalign without coupling score (independent-site PPalign) achieves an $F_1$ score comparable to HHalign (0.580 vs 0.578) despite a poor handling of gaps by Potts models as opposed to pHMMs. Besides, while PPalign’s alignment is most of the time better with the coupling score, 2 sequence pairs were yet significantly better aligned by independent-site PPalign than by PPalign with couplings: on already discussed AL10050875, where it improves a bit the poor quality of the alignment by PPalign, but also on AL00089447 ($12.93\%$ sequence identity) where it improves over the improvement of HHalign on PPalign.

## Discussion

Although the problem is very likely to be NP-hard since the threading problem is NP-hard [38], these experiments demonstrate that PPalign yields optimal Potts to Potts alignments up to a precision $\epsilon$ in tractable time. These results have to be confirmed on bigger instances. For now, experimentation is limited by memory handling in CCMpredPy, which is currently the only inference method offering the features we require to infer comparable Potts models, but the current implementation of CCMpred [30] shows that this type of inference can be optimized to handle significantly larger models. This should enable us to test larger alignments in the future. Based on our experimentation, we expect these alignments to be also tractable. This is surprising with respect to the NP-complete nature of the problem, but it seems that alignments of Potts models are not the hardest instances when they properly represent homologous proteins. We think that this depends yet on the choice of the parameters shaping the inference of Potts models and the similarity of the models to align: these questions deserve further studies to better understand the application scope of this method.

Regarding alignment quality, our results for the alignment of Potts models inferred using a pseudo-likelihood method designed for co-evolution prediction purposes are overall better than for the alignment of pHMMs by HHalign, with significant examples demonstrating how taking couplings into account can improve the alignment of remote homologous proteins, especially for lowest similarity alignments. There is still room for improvement in our method. We have noticed a tendency to align too many positions that can be corrected and our worst score with respect to HHalign is associated with very gappy train MSAs, indicating that augmenting Potts models with an appropriate gap handling strategy would undoubtedly improve our results. Above all, it is worth noting that independent-site PPalign finds sometimes a better alignment than PPalign, coupling matrices bringing more noise than assistance in these cases. To get better alignments, the priority is now is to work on more robust inference of Potts models, to make them more comparable and informative for homology search despite the relatively small size of training samples. We proposed here some ideas towards the inference of more canonical Potts models, with only the necessary couplings, as well as some post-processing steps, notably to smooth weights by simulated uniform pseudocounts. This later step allowed us to raise the average $F_1$ score from 0.48 to 0.60, but we think that a more direct procedure would still be preferable. We are now searching for an efficient Potts model inference method that can be geared towards canonicity, providing the possibility to add pseudo-counts on the single and double amino acid counts – thus excluding methods based on pseudo-likelihood maximization – and being able to infer extended Potts models with an appropriate gap handling strategy. Besides, though the focus of this paper was the alignment of models inferred on pre-built multiple sequence alignments, it should be noted that the quality of the sequence alignments we provide strongly depends on the quality of their associated MSAs. The use of a suitable inference method will allow us to properly test this dependency on the method used to retrieve close homologs and on the chosen alignment depth in future experiments.

## Conclusion

While Potts models have been successfully used for contact prediction and other tasks on protein sequences, using coevolutionary information captured by direct coupling analysis to improve homology search by sequence alignment seems promising, but challenging. The main computational bottleneck is the hardness of alignments involving Potts models.

We presented here PPalign, our method for Potts model to Potts model alignment based on the introduction of an Integer Linear Programming formulation of the problem with an implementation relying on an efficient solver able to yield the optimal solution in tractable time. This initiates a new approach for remote homology search by alignment of Potts models inferred from close homologs, similarly to HHalign with the alignment of pHMMs but with the addition of long distance sequence correlations reflecting the 3D structure of proteins. In this approach, Potts models need to be comparable. As a basic principle for building canonical Potts models, we proposed to infer models with as much weight as possible on the positional parameters and to add only necessary weight on pairwise couplings. We also proposed a scheme for lessening the effects of small sample variations on the Potts model’s parameters.

To experimentally assess the feasibility and interest of the approach, we carefully selected a set of non-redundant reference pairwise alignments with low sequence identity and with enough close homologs for each aligned sequence to infer a Potts models. We carried out rigorous experimentation with a strict separation of data used to train hyperparameters of the method and data used to test its performances. Results on test alignments confirm that Potts models can be aligned in reasonable time ($1'37''$ in average) and that taking into account direct coupling information can improve sequence alignments, especially for remote homologs with lowest sequence identity.

Our experiments suggest that new research on the inference of Potts models could improve their usefulness for homology search. The approach would undoubtedly benefit from extending to Potts models the insertion/deletions modeling capacities as well as the efficient pseudocount schemes of pHMMs. Maybe a more difficult issue is to have guarantees on a canonical form or at least some robustness of inferred Potts models to make them more comparable. We hope that PPalign’s efficiency and optimality will help to perform unbiased investigations in these directions.

## Data Availability

Software and data are available here: https://www-dyliss.irisa.fr/ppalign

## Abbreviations

MSA: Multiple sequence alignment
pHMM: Profile Hidden Markov Model
ILP: Integer Linear Programming
UB: Upper bound
LB: Lower bound
